# An integrative approach to detect epigenetic mechanisms that putatively mediate the influence of lifestyle exposures on disease susceptibility

**DOI:** 10.1093/ije/dyz119

**Published:** 2019-06-29

**Authors:** Tom G Richardson, Rebecca C Richmond, Teri-Louise North, Gibran Hemani, George Davey Smith, Gemma C Sharp, Caroline L Relton

**Affiliations:** MRC Integrative Epidemiology Unit (IEU), Population Health Sciences, Bristol Medical School, University of Bristol, Oakfield House, Oakfield Grove, Bristol, UK

**Keywords:** DNA methylation, Mendelian randomization, mediation, smoking, ALSPAC, ARIES

## Abstract

**Background:**

There is mounting evidence that our environment and lifestyle has an impact on epigenetic regulatory mechanisms, such as DNA methylation. It has been suggested that these molecular processes may mediate the effect of risk factors on disease susceptibility, although evidence in this regard has been challenging to uncover. Using genetic variants as surrogate variables, we have used two-sample Mendelian randomization (2SMR) to investigate the potential implications of putative changes to DNA methylation levels on disease susceptibility.

**Methods:**

To illustrate our approach, we identified 412 CpG sites where DNA methylation was associated with prenatal smoking. We then applied 2SMR to investigate potential downstream effects of these putative changes on 643 complex traits using findings from large-scale genome-wide association studies. To strengthen evidence of mediatory mechanisms, we used multiple-trait colocalization to assess whether DNA methylation, nearby gene expression and complex trait variation were all influenced by the same causal genetic variant.

**Results:**

We identified 22 associations that survived multiple testing (*P* < 1.89 × 10^–7^). In-depth follow-up analyses of particular note suggested that the associations between DNA methylation at the *ASPSCR1* and *REST/POL2RB* gene regions, both linked with reduced lung function, may be mediated by changes in gene expression. We validated associations between DNA methylation and traits using independent samples from different stages across the life course.

**Conclusion:**

Our approach should prove valuable in prioritizing CpG sites that may mediate the effect of causal risk factors on disease. In-depth evaluations of findings are necessary to robustly disentangle causality from alternative explanations such as horizontal pleiotropy.

**Key Messages**

- Epigenetic factors, such as DNA methylation, are biological processes that may be responsible for mediating the effect of lifestyle exposures on disease outcomes.
- Mendelian randomization and multiple-trait colocalization can be used to assess potential consequences of adverse risk factors for disease susceptibility due to changes in DNA methylation levels.
- In this study, we have demonstrated the value of our analytical pipeline by assessing the downstream effects of prenatal smoking on 643 different complex traits.
- Our strongest evidence of association was observed at gene regions linked with reduced lung function, which we evaluated using two independent populations.
- Future projects that harness increasingly large-scale molecular data should help us disentangle complex mechanisms in epidemiology.

## Introduction

Epigenetic factors have long been postulated as molecular mechanisms that are responsible for mediating the effect of causal risk factors on disease outcomes.^1–4^ However, evidence implicating specific mechanisms across the genome in this regard is scarce. Recent epigenome-wide association studies (EWAS) have identified hundreds of associations between risk factors and variation in DNA methylation levels^5–8^, an epigenetic regulation process known to play a key role in cell development and disease susceptibility.^9^^,^^10^ Interpreting the findings of EWAS is a challenging endeavour, particularly due to reverse causation and unmeasured confounding factors that may influence results.^11–13^

An approach that can be used to address this challenge is Mendelian randomization (MR), a method by which genetic variants can be used as a proxy for modifiable exposures to infer causality among correlated traits.^14–16^ Research undertaken using the Accessible Resource for Integrated Epigenomics Studies (ARIES) resource^17^ has identified thousands of CpG sites where variation in DNA methylation levels is associated with genetic variants, known as methylation quantitative trait loci (mQTL).^18^ Undertaking MR analyses using mQTL as instrumental variables can therefore be used to investigate potential downstream effects of changes in DNA methylation levels on human health and disease risk. Furthermore, under the assumptions of two-sample MR (2SMR), it is possible to harness findings from large-scale, well-powered genome-wide association studies (GWAS) to obtain effect estimates for mQTL on hundreds of complex traits across the human phenome.

**Figure 1.**
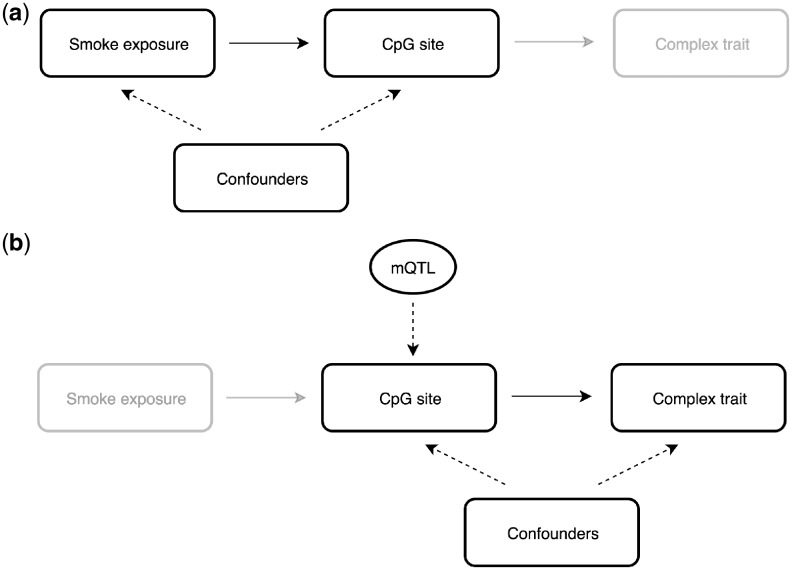
An overview of the proposed two-step epigenetic Mendelian randomization approach to evaluate findings from epigenome-wide association studies. (a) Identify CpGs sites from EWAS where a risk factor is associated with DNA methylation. Prenatal risk factors can add value as they are very unlikely to be associated due to reverse causation. (b) Use independent *cis*-acting methylation quantitative trait loci (mQTL) as an instrumental variable to proxy for changes in DNA methylation levels at this CpG site, allowing investigation into how these effects may influence complex traits.

We have undertaken a study that builds upon the methodology established by two-step epigenetic MR^19^ to assess the potential downstream effects of DNA methylation variation associated with smoking^5^ (Figure 1). We focus on this exposure as a key risk factor to illustrate this approach as previous studies have provided strong evidence that it acts as a determinant of DNA methylation levels.^20–22^ Moreover, large EWAS of prenatal smoking have identified hundreds of CpG sites across the genome associated with DNA methylation levels, which is attractive when investigating consequences of epigenetic changes as reverse causation is extremely unlikely (i.e. prenatal smoke exposure influences DNA methylation and not vice versa). We have investigated consequences of these putative molecular changes across the human phenome using findings from 643 large-scale GWAS. Next we applied multiple-trait colocalization to discern whether variation in DNA methylation levels may influence traits via changes in the transcription of nearby genes to associated CpG sites. Finally, we have undertaken sensitivity analyses to explore associations in detail, as well as validation analyses using an independent sample of young individuals to investigate potential *in utero* effects.

## Methods

### Study population

The Avon Longitudinal Study of Parents and Children (ALSPAC) is a population-based cohort study investigating genetic and environmental factors that affect the health and development of children. The study methods are described in detail elsewhere^23^^,^^24^ (http://www.bristol.ac.uk/alspac). Briefly, 14 541 pregnant women residents in the former region of Avon, UK, with an expected delivery date between 1 April 1991 and 31 December 1992, were eligible to take part in ALSPAC. Detailed information and biosamples have been collected on these women and their offspring at regular intervals, and are available through a searchable data dictionary (http://www.bris.ac.uk/alspac/researchers/our-data/). Written informed consent was obtained for all study participants. Ethical approval for the study was obtained from the ALSPAC Ethics and Law Committee and the Local Research Ethics Committees.

DNA methylation used in the study was derived using blood samples obtained from 1018 ALSPAC mother–offspring pairs (mothers at two timepoints and their offspring at three timepoints) as part of the Accessible Resource for Integrated Epigenomic Studies (ARIES) project.^17^ The Illumina HumanMethylation450 (450 K) BeadChip array was used to measure DNA methylation at over 480 000 sites across the epigenome. Genotype data were available for all ALSPAC individuals enrolled in the ARIES project, which had previously undergone quality control, cleaning and imputation at the cohort level. Details on methylation assays, genotyping and imputation can be found in the Supplementary Material, available as Supplementary data at *IJE* online.

### Identifying eligible CpG sites using results from EWAS and the mQTL database

Prior to undertaking analysis, we identified CpG sites across the epigenome that current findings and accessible datasets allowed us to investigate. As an example, we obtained a list of 2303 CpG sites that were associated with prenatal smoking based on findings from Joubert *et al*.,^5^ using a false discovery rate (FDR)<0.01 in their analysis adjusted for cell counts. Next, we evaluated which of these CpGs we were able to instrument using an mQTL. In this study we used the mQTL database (http://www.mqtldb.org) for this purpose, which reports SNP-methylation effect estimates obtained from the ARIES project (http://www.ariesepigenomics.org.uk/).^17^^,^^18^ However, there will likely be alternative resources to this with larger sample sizes in the forthcoming years. Moreover, future research may eventually be able to access mQTL effects derived from different tissue types than whole blood as undertaken in this study.

As we wanted to use mQTLs as instrumental variables for changes in DNA methylation influenced by prenatal smoke exposure, we have only used results from the birth timepoint in ARIES (i.e. based on DNA methylation data derived from cord blood). Conditionally independent mQTLs were identified in ARIES using the COJO-slct routine in GCTA^25^^,^^26^ and applying a threshold of *P* < 1.0 × 10^–7^. This threshold was merely applied as a heuristic to identify instruments for our analysis though and future applications may wish to apply alternative criteria. Furthermore, we only identified *cis*-mQTLs as suitable instrumental variables for our analysis, i.e. genetic variants associated with proximal DNA methylation levels (<1 MB distance away). This is because *trans*-mQTL are more likely to be prone to horizontal pleiotropy, i.e. where the genetic variant influences DNA methylation and the associated complex trait via two independent biological pathways. As CpG sites in ARIES are typically only independently associated with a single *cis*-acting mQTL, this was an important consideration as we anticipated that downstream analyses would be unable to robustly investigate horizontal pleiotropy. However, future resources that uncover a larger number of conditionally independent mQTL to instrument CpG sites may contemplate their inclusion in study designs.

### Complex trait data from large-scale GWAS and the UK Biobank study

We next obtained observed effects for each mQTL identified in the previous stage of our analysis on a range of complex traits using large-scale studies available from the MR-Base platform (http://www.mrbase.org).^27^ The following inclusion criteria were used to select complex traits to be analysed:

- Study samples must be larger than 1000
- Effects reported genome-wide for over 100 000 genetic variants
- Either European or mixed populations
- Reported beta, standard error (SE) and effect alleles for variants

We also downloaded GWAS summary statistics from the Neale lab^28^ for complex traits not represented in MR-Base. When effect estimates for complex traits were not available for an mQTL, we identified a proxy instrument in high linkage disequilibrium (*r*^2^ > 0.8) using genotyped data from Europe individuals as part of phase 3 (version 5) of the 1000 genomes project.^29^ Although we have evaluated associations on a wide array of traits in this study, future applications may wish to investigate hypothesis-driven relationships depending on the environmental exposure of interest.

### Statistical analysis

#### Two-sample Mendelian randomization

We used 2SMR to evaluate whether variation in DNA methylation at CpG sites associated with our exposure (i.e. prenatal smoke exposure in this study) were also associated with complex trait variation (Figure 1). When DNA methylation levels at CpG sites only had a single *cis*-acting instrument (i.e. independent *cis*-mQTL), MR analyses were undertaken using the Wald ratio test.^30^ For single genetic variant *j*, this can be calculated by taking the ratio of the gene–outcome association (denoted by $\hat{\Gamma }j$) and the gene–exposure association (denoted by $\hat{\gamma }j$) estimates^31^:

$${\hat{\beta }}_{j}=\frac{{\hat{\Gamma }}^{j}}{{\hat{\gamma }}^{j}}$$

Where two or more genetic instruments were available for a CpG site, we used the inverse variance weighted (IVW) method to obtain MR effect estimates^32^:

$${\hat{\beta }}_{\mathrm{IVW}}=\;\frac{{{{\sum_{j}\hat{\gamma }}_{j}^{2}\sigma }_{\mathrm{Yj}}^{-2}{\hat{\beta }}_{j}}_{\;}}{{{\sum_{j}\hat{\gamma }}_{j}^{2}\sigma }_{\mathrm{Yj}}^{-2}}$$

where $\sigma_{\mathrm{Yj}}$ is the SE of the gene–outcome estimate for variant *j*. Although the majority of CpG sites in our study could only be instrumented using a single genetic variant, we expect the number of independent mQTL for a given CpG site to increase with larger sample size mQTL studies. As such, future applications of our approach may benefit from using the IVW method to harness multiple independent *cis*-acting variants as instrumental variables. Furthermore, this will allow MR sensitivity analyses to be undertaken, such as assessing heterogeneity and conducting leave-one-out analyses.

#### Multiple-trait colocalization

When investigating how changes in DNA methylation may influence downstream traits in an MR framework, it is important to evaluate whether association signals may be a product of linkage disequilibrium between two separate causal variants (i.e. one influencing changes in DNA methylation and another influencing trait variation).^33^^,^^34^ Furthermore, if changes in DNA methylation at a CpG site truly influence complex trait variation, then they are likely to be doing so via changes in the expression of a nearby gene. We therefore applied multiple-trait colocalization^35^ to investigate associations that survived multiple testing comparisons in the 2SMR analysis (i.e. 0.05/number of CpG-trait combinations analysed). This allowed us to assess whether the genetic variant responsible for changes in DNA methylation is also responsible for both variation in nearby gene expression and the associated complex trait.

Multiple-trait colocalization was applied using mQTL data from the Focus on Mother’s (FOM) timepoint in ARIES (mean age: 47.5), GWAS summary data for the associated complex trait and expression quantitative trait loci (eQTL) data derived from whole blood that was obtained from the GTEx consortium v7 (*n* = 369).^36^ mQTL data from the FOM timepoint was used to evaluate whether effects observed in the previous analysis using cord blood persisted until later life. We ran this analysis multiple times to systematically investigate colocalization with the expression of all genes within 100 kb of the CpG site of interest. Analyses were only undertaken if there were at least 50 variants (minor allele frequency ≥ 5%) in common between all three datasets. As recommended by the authors of this approach, a posterior probability of association (PPA) of 80% or higher was considered evidence of colocalization. Additionally, multiple testing was not accounted for in these analyses as the authors of the multiple-trait colocalization paper demonstrated that after 500 000 simulations there was no indication of inflated false-positive rates using this method (even with a PPA threshold of 30%). If a CpG site was associated with multiple correlated traits, only the trait with the highest PPA in the colocalization analysis was taken forward.

#### Orienting directions of effect between traits

For associations that provided evidence of colocalization in the previous analysis, we evaluated the direction of effect between 2SMR estimates and observed effects from the Joubert *et al*. study.^5^ This allowed us to orient direction of effects between prenatal smoke exposure, DNA methylation and the complex traits. Such evaluations are important to validate that directions of effect between environmental exposures and outcomes are consistent with evidence from the literature.

We also undertook an analysis in ALSPAC to investigate the association between maternal smoking and any traits with evidence of association from our MR and multiple-trait colocalization analysis. This was to evaluate the proportion of the effect mediated via changes in DNA methylation levels along the causal pathway between our exposure and associated outcome. Analyses in this study were adjusted for maternal education, age and sex.

To investigate evidence of reverse causation, we evaluated the association between complex traits (i.e. modelled as our exposure) and DNA methylation (i.e. our outcome) in a 2SMR framework. As a sensitivity analysis, we also used the MR directionality test to assess the direction of causality between molecular and complex traits.^37^ This test was applied three times for each association, to evaluate the direction between each pair of traits (i.e. DNA methylation, gene expression and complex trait). This allowed us to undertake an exploratory analysis regarding the chain of direction between all three traits concerned.

#### Comparison of findings using two independent samples

We also repeated 2SMR analyses for the associations that provided evidence of colocalization using two independent samples. Along with validating findings in separate populations, this stage of our approach can be valuable in terms of exploring the temporality of associations. For instance, as we selected prenatal smoking as our exposure in this study, we were interested in assessing the effect estimates for findings at an early stage in the life course.

For this we undertook an initial analysis within the ALSPAC cohort, using mQTL effects from ARIES participants during childhood (mean age: 7.49 years) and effects on complex traits from ALSPAC individuals not enrolled in the ARIES project [at either the age 7, age 9 or teen focus 3 (mean age 15.5 years) clinics]. Analyses were then repeated using ARIES participants from the adolescence time point (mean age: 15.5 years). This allowed us to investigate the potential confounding effect of own smoke exposure that is more likely to be a factor at age 15 rather than age 7. If observed associations are due to *in utero* effects from maternal smoke exposure, then we may expect them to be observed consistently at both timepoints. However, this analysis cannot completely rule out own smoke exposure as a confounding factor, nor alternative explanations such as offspring having shared genetics and other environmental exposures as their parents.

The second analysis was undertaken using mQTL effects for the same CpG sites using results from the BIOS QTL browser^38^ and complex trait data from large-scale GWAS and/or the UK Biobank study^27^^,^^28^ to determine MR effect estimates in adulthood. Further publicly accessible resources such as these in the forthcoming years should prove beneficial for the validation stage of our approach.

Finally, to further evaluate any potential *in utero* effects, we repeated our initial analysis at associated CpG sites in the UK Biobank study.^39^ Here we stratified the sample based on whether individuals answered yes or no to the question ‘Did your mother smoke regularly around the time when you were born?’. Stratified analyses can be insightful in terms of better understanding putative epigenetic effects, such as investigating the impact of prenatal exposures in our study. However, they may require individual-level data to undertake and as such this step of our study may not always be possible depending upon the accessibility of data.

All analyses were undertaken using R (version 3.31). Plots illustrating multiple trait colocalization were generated using base R graphics. Code used in this project can be found at https://github.com/eptgr/prenatal-CpGs. Power calculations based on the effect sizes identified in this study were undertaken using the MR power calculator.^40^

## Results

### Assessing the impact of DNA methylation variation at birth on the human phenome

There were 412 unique CpG sites associated with prenatal smoke exposure (FDR<0.01)^5^ that met our inclusion criteria based on data from the ARIES project^17^ (Figure 1a, Supplementary Table 1, available as Supplementary data at *IJE* online). We then assessed the potential effect of changes in DNA methylation on 643 complex traits at these CpG sites using 2SMR (Figure 1b, Supplementary Table 2, available as Supplementary data at *IJE* online).

**Figure 2.**
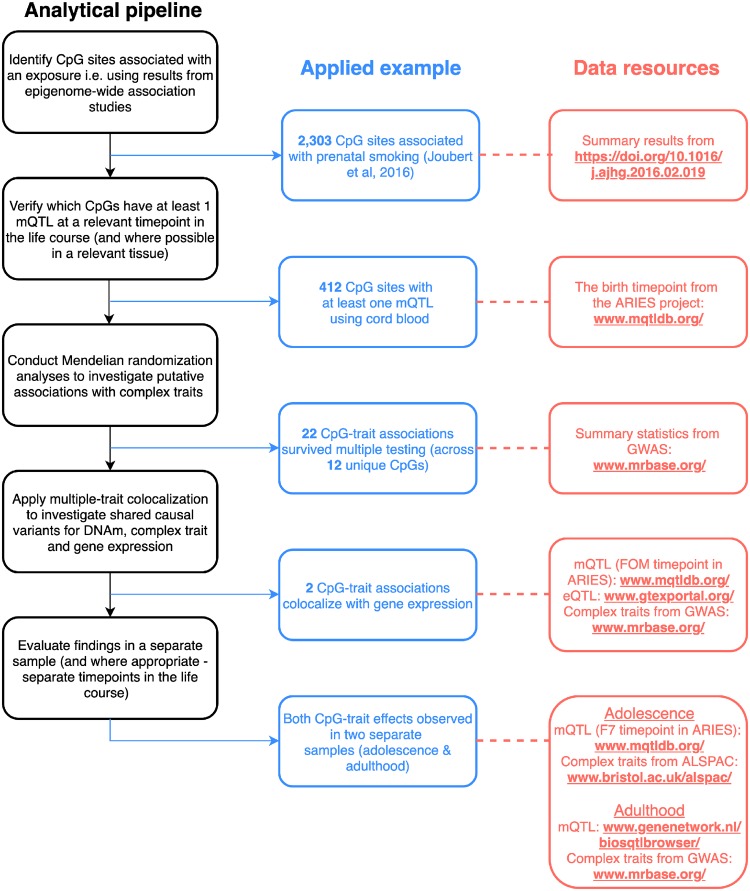
Flowchart outlining the analysis pipeline used in this study along with findings from our applied example and the data resources used. mQTL, methylation quantitative trait loci; DNAm, DNA methylation; ARIES, accessible resource for integrated epigenomics studies; GWAS, genome-wide association studies; eQTL, expression quantitative trait loci; ALSPAC, Avon Longitudinal Study of Parents and Children.

Overall there were 22 CpG-trait associations across 12 distinct CpG sites that survived multiple testing corrections (*P* < 1.89 × 10^−^^7^) (Table 1 and Supplementary Table 3, available as Supplementary data at *IJE* online). Among these associations were measures of anthropometry (height and waist-hip-ratio), lung function [forced vital capacity and forced expiratory volume in 1 second (FEV1)], cardiovascular traits (low-density lipoprotein cholesterol and systolic blood pressure) and bone mineral density. A flowchart illustrating the analysis pipeline used in this study to identify and evaluate these effects can be found in Figure 2.

**Table 1.** Top findings from Mendelian randomization analysis for prenatal smoke exposure-associated CpG sites and complex traits

CpG site	Gene	Complex trait	MR Beta (SE)	MR *P*	PPA_abc_
cg26930078	*C17orf53*	BMD	0.093 (0.010)	1.50 × 10^−20^	1.84 × 10^−05^
cg02812767	*LOXL1*	FVC	−0.051 (0.006)	3.78 × 10^−20^	0.220
cg08685733	*C17orf53*	BMD	−0.108 (0.013)	5.78 × 10^−18^	0.176
cg25313468	*REST*	Height	−0.064 (0.008)	5.37 × 10^−17^	9.61 × 10^−06^
cg06105699	*ASPSCR1*	FEV1	0.053 (0.007)	7.29 × 10^−15^	**1**
cg23184042	*DLX6AS*	BMD	−0.038 (0.005)	1.26 × 10^−14^	1.31 × 10^−04^
cg14150774	*QSOX2*	FVC	0.038 (0.005)	1.99E−12	5.31 × 10^−05^
cg18883198	*TMEM57*	LDL	−0.099 (0.016)	2.72 × 10^−10^	5.22 × 10^−06^
cg01401641	*TSHZ3*	SBP	0.040 (0.007)	4.25 × 10^−09^	0.158
cg01307174	*ARPP-21*	Worrying	0.025 (0.004)	2.65E−08	0.003
cg25313468	*REST*	FEV1	−0.030 (0.006)	4.86 × 10^−8^	**0.955**
cg18089426	*DLK1*	Age at menarche	0.099 (0.019)	9.00 × 10^−08^	1.09 × 10^−04^
cg06070002	*PRDX1*	Waist-to-hip ratio	0.056 (0.011)	1.08 × 10^−07^	9.62 × 10^−05^

MR Beta (SE) units are in standard deviations (SD) meaning that 1 SD increase in DNA methylation relates to *X* SD change in the trait. CpG site, ID based on Illumina mappings; Gene, as reported by Joubert *et al.*^5^ or nearest gene if not specified; MR, Mendelian randomization; SE, standard error; *P*, P-value; PPA_abc__,_ highest posterior probability of association of colocalization between all 3 traits i.e. DNA methylation (a), gene expression (b) and complex trait (c) (PPAs surviving the 0.8 threshold used in this study are in bold). BMD, bone mineral density; FVC, forced vital capacity; FEV1, forced expiratory volume in 1 second; LDL, low-density lipoproteins; SBP, systolic blood pressure; Worrying, a Yes/No questionnaire based outcome in response to the question ‘Do you worry too long after embarrassment?’

### Elucidating putative epigenetic mechanisms in disease using multiple-trait colocalization

**Figure 3.**
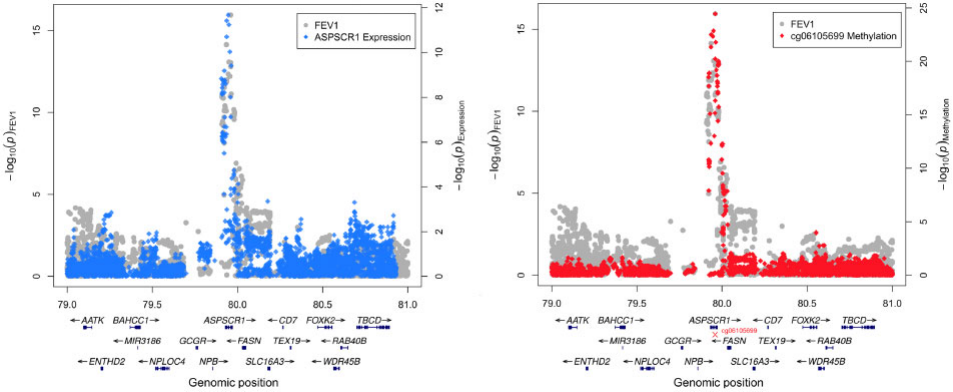
Illustration of genetic colocalization at the *ASPSCR1* locus between gene expression and lung function (left) as well as DNA methylation at CpG site cg06105699 and lung function (right). Overlapping distributions of –log_10_*P*-values for the effects of genetic variants at the *ASPSCR1* gene region on forced expiratory volume in 1 second (FEV1) (grey), gene expression of *ASPSCR1* in whole blood (blue) and DNA methylation at cg06105699 (red). Genes at this region are annotated below the *x*-axis, where a red cross indicates the position of the associated CpG site. Multiple-trait colocalization provides strong evidence that FEV1, *ASPSCR1* expression and DNA methylation at cg06105699 all share the same underlying causal variant at this locus. This supports evidence that changes in DNA methylation at this CpG site may influence lung function via changes in gene expression.

If DNA methylation levels at a CpG site truly have an impact on human health and disease, then we would expect the transcription of nearby genes to also be involved. Therefore, to strengthen evidence of a causal association, we integrated data on gene expression from the GTEx consortium^36^ for all genes within a 100 kb distance of the CpG sites associated with complex traits. As DNA methylation data in this study was only available using data from whole blood, we only used gene expression data also derived from this tissue type from GTEx. Applying multiple-trait colocalization provided evidence at two CpG sites that variation in gene expression, DNA methylation and complex traits were driven by the same underlying genetic variant (Supplementary Table 4, available as Supplementary data at *IJE* online). These were cg06105699 and cg25313468 which are located at the promoter regions of the *ASPSCR1* and *REST* genes respectively. DNA methylation at cg06105699 colocalized with measures of lung function and the expression of *ASPSCR1* (highest PPA = 1), whereas cg25313468 colocalized with measures of lung function and the expression of *POL2RB* (highest PPA = 0.95). Figure 3 illustrates the overlapping distributions of effects for genetic variants at the *ASPSCR1* region on FEV1, *ASPSCR1* expression and DNA methylation at cg06105699. Supplementary Figure 1, available as Supplementary data at *IJE* online, provides a similar plot illustrating the effect at the *REST*/*POL2RB* locus. There was also evidence of colocalization at one other CpG site for DNA methylation and complex traits but not gene expression (cg08685733 near *C17orf53*, associated with bone mineral density) (Supplementary Table 5, available as Supplementary data at *IJE* online).

### Orienting the direction of effect between prenatal smoke exposure, molecular traits and outcomes

Using observed estimates reported by the EWAS of prenatal smoking^5^ we were able to infer that prenatal smoke exposure was putatively associated with reduced lung function via methylation at the *ASPSCR1* and *REST/POLR2B* loci. Prenatal smoke exposure was negatively associated with DNA methylation at *ASPSCR1* [Beta: −0.008 (SE: 0.002)] and our MR analysis suggests that reduced DNA methylation at this CpG site correlates with reduced lung function [MR Beta: 0.053 (MR SE: 0.007)]. At the *REST/POLR2B* locus, prenatal smoke exposure was positively associated with DNA methylation levels [Beta: 0.012 (SE: 0.003)] and our MR analysis suggests that increased methylation at this CpG site also may influence reduced lung function [MR Beta: −0.030 (MR SE: 0.006)].

We next undertook MR analyses to evaluate reverse causation (i.e. whether genetic liability to variation in lung function has an effect on DNA methylation at these CpG sites), although there was no strong evidence of this (lowest *P* = 0.026 across 20 tests) (Supplementary Table 6, available as Supplementary data at *IJE* online). A sensitivity analysis using the MR directionality test complemented these findings, as it suggested the direction of effect between molecular and complex traits at each locus was that DNA methylation influences gene expression, which subsequently influences lung function (Supplementary Table 7, available as Supplementary data at *IJE* online). Although we may hypothesize that this chain of events is the most parsimonious explanation for associations based on the results of our study, in-depth function studies are necessary to robustly demonstrated that this is the true underlying causal pathway.

Our analysis with regard to maternal smoking and FEV1 in ALSPAC identified weak evidence of association (Beta = −0.02, SE = 0.01, *P* = 0.07, *n* = 5417). However, the direction of effect in this analysis was consistent with oriented effects between prenatal smoke exposure, CpGs and FEV1 (i.e. maternal smoking is associated with reduced lung function). We were however unable to investigate the proportion of effect potentially mediated between our exposure and outcome via changes in DNA methylation in this analysis. Future studies interested in applying our approach in larger samples sizes may benefit from investigating the proportion potentially mediated along putative causal pathways using well-powered effect estimates.

### Validation of results using multiple timepoints over the life course

We investigated the observed associations at the two CpG sites of interest during early stages in the life course using data from the ALSPAC cohort.^23^ There was weak evidence that DNA methylation levels at either CpG site was associated with FEV1 at age 8 (*ASPSCR1*—MR Beta (SE) = −0.017 (0.010), *P* = 0.086, *n*_outcome_ = 5552; *REST/POL2RB*—MR Beta (SE)  = 0.014 (0.012), *P* = 0.226, *n*_outcome_ = 4644). However, evidence was stronger at age 15.5 despite having a smaller sample size (*ASPSCR1*—MR Beta (SE) = −0.073 (0.029), *P* = 0.013, *n*_outcome_ = 3852; *REST/POL2RB*—MR Beta (SE) = 0.078 (0.032), *P* = 0.016, *n*_outcome_ = 3240). Full results from this analysis can be found in Supplementary Table 8, available as Supplementary data at *IJE* online. As smoking initiation is likely to be more prevalent at age 15 rather than age 8, these findings support a role for own smoking (or environmental smoke exposure) influencing lung function via changes in DNA methylation levels, as opposed to an *in utero* effect due to maternal smoke exposure. Our split sample MR analysis using data from the UK Biobank supported this, as the observed associations were not confined to the subset of individuals who reported exposure to maternal smoking around birth (Supplementary Table 9, available as Supplementary data at *IJE* online).

We also evaluated these effects using data obtained during adulthood that provided strong evidence of association between DNA methylation at these loci and their relevant traits (*ASPSCR1*—MR Beta (SE) = −0.025 (0.005), *P* = 9.23 × 10^–08^, *n*_outcome_ = 307 638; *REST/POL2RB*—MR Beta (SE) = 0.034 (0.005), *P* = 2.90 × 10^−^^13^, *n*_outcome_ = 307 638). This was undertaken in an independent sample of individuals to the analysis in adolescence, as we used mQTL data from the BIOS QTL browser^38^ and the UK Biobank study.^39^ These findings therefore help validate the robustness of these associations, as well as suggesting that own smoke exposure may be more likely to be responsible for putative effects rather than maternal smoke exposure. Moreover, the direction of MR effects at both loci were consistent between the population of adults and adolescents (Supplementary Table 8, available as Supplementary data at *IJE* online).

## Discussion

In this study we have proposed a pipeline of analytical techniques to investigate the potential functional implications of associations between lifestyle risk factors and measures of DNA methylation. We have illustrated this approach by evaluating CpG sites previously linked with maternal smoke exposure, where the strongest evidence of association suggested that putative smoke exposure-associated epigenetic changes may potentially influence reduced lung function at the *ASPSCR1* and *REST/POL2RB* loci via changes in gene expression. The other association signals detected by this study require further validation to robustly link DNA methylation variation with later life outcomes, although upcoming datasets across various tissue types will facilitate such endeavours. Furthermore, this proposed pipeline should prove valuable for future studies that harness these forthcoming datasets and findings of large-scale EWAS. Doing so will help uncover putative epigenetic mechanisms that may play a mediatory role between risk factors and disease.

DNA methylation variation at either the *ASPSCR1* or *REST/POL2RB* locus has not previously been associated with measures of lung function by published EWAS.^41^^,^^42^ A possible explanation for this could be the reasonably modest sample sizes analysed in these studies (*n* = 100 and *n* = 172, respectively), which may be attributed to the current costs of DNA methylation sequencing assays. This highlights the strength of our pipeline in terms of leveraging summary statistics from a large-scale consortium in a 2SMR framework, thus circumventing the requirement of having DNA methylation and trait measurements in the same population. Furthermore, this allows our pipeline to evaluate associations with a wide array of traits, which again would be challenging in a one-sample setting. We note however that as larger sample sizes become available for EWAS it would be valuable to compare observational effect estimates with findings from MR analyses. This is particularly important given that observational estimates may be biased and disguise a true underlying relationship.

There was an inverse relationship between prenatal smoke exposure and lung function potentially mediated by epigenetic variation at *ASPSCR1* and *REST/POL2RB*, which is in line with evidence from the literature concerning exposure to tobacco smoke early in the life course and later-life lung function.^43^^,^^44^ We also observed consistent directions of effect and associations between DNA methylation and lung function at these loci in two separate populations. Interestingly, the association observed using individuals from the ALSPAC cohort was stronger when using measures of lung function from the age 15 timepoint compared with the age 8 one, despite having a smaller sample size (*n* = 3852 and 3240 vs *n* = 5552 and 4644 respectively). This implies that these associations are unlikely to be attributed to an *in utero* effect due to maternal smoke exposure. Furthermore, it may be that the most parsimonious explanation for these results is that both genetics and risk factors such as prenatal smoke exposure collectively influence traits via changes in DNA methylation. That being said, this approach should still prove useful for future studies interested in identifying *in utero* effects, although as demonstrated by this study we advise using a cohort of young individuals to evaluate findings. Identifying specific epigenetics mechanisms that can explain *in utero* effects is a challenging but potentially extremely rewarding area of research.^45^

The association signal we detected in this study at cg25313468 corresponds to a CpG site that resides near the promoter region of the *REST* gene. However, the strongest evidence of co-localization was detected with the expression of the neighbouring *POL2RB* gene, along with DNA methylation at this CpG site and FEV1. The functional gene responsible for this putative association is therefore unclear, as is whether there may be in fact multiple genes at this locus whose transcription may be influenced by potential changes in DNA methylation due to prenatal smoke exposure. The *REST* gene encodes the RE1-silencing transcription factor that has been reported to be a transcriptional repressor.^46^^,^^47^ Although this has been principally linked to neuronal genes in non-neuronal tissues,^47^ there is also previous evidence suggesting that *REST* functions as a master regulator of gene repression in response to hypoxia (deprivation of adequate oxygen reaching tissues).^48^ There is also previous evidence that maternal RE1-silencing transcription factor regulates the expression of 158 genes, which may have implications in both development and long-term phenotypes.^49^ In contrast, evidence implicating the *POL2RB* gene at this locus is less clear, although epigenetic regulation of *POL2RA* has been previously linked with childhood wheezing due to maternal stress.^50^

At the other CpG site with evidence of association in this study, concerning DNA methylation at cg06105699, there was evidence of colocalization with two nearby genes (*ASPSCR1* and *LRR45*). *ASPSCR1* is a candidate gene for alveolar soft part sarcoma (ASPS), a rare type of soft tissue sarcoma that typically occurs in young patients and often spreads to the lungs, brain and bone.^51^ There is evidence to suggest that this is due to oncogenic rearrangement with the transcription factor gene *TFE3.*^52^ The function of *LRRC45* is less clear, although it is known to play a role in centrosome cohesion.^53^ Further analyses are therefore required to separate co-expression between these proximal genes to better understand the potential effect at this locus.

Our proposed split sample analysis using UK Biobank data should prove useful for future studies wishing to evaluate putative associations for certain exposures, such as prenatal smoking that was investigated in this study. Furthermore, exploring associations between an exposure and outcome across multiple timepoints can enhance understanding of temporal effects. In this study we undertook these types of analyses to demonstrate that associations are more likely to be attributed to own smoking (or possibly environmental smoke exposure) rather than an *in utero* effect. However, we appreciate that the feasibility of undertaking these analyses in future studies may depend on the accessibility of appropriate datasets in large sample sizes. As such, design choices should be carefully considered before applying our pipeline to investigate a research question. Longitudinal cohorts such as ALSPAC can be extremely useful in terms of exploring the temporality of effects, whereas richly phenotyped resources such as UK Biobank are powerful for independent validation. We also undertook some power calculations based on the effect sizes observed in our own study regarding lung function traits (which typically ranged from 0.03 to 0.05 standard deviation increase in outcome per standard deviation change in exposure). These results suggest that harnessing summary statistics from biobank-scale datasets considerably helps in achieving adequate statistical power for the types of analyses proposed in our study (Supplementary Figure 2, available as Supplementary data at *IJE* online).

A limitation of this study is the lack of tissue-specific data at birth, which is why we have used DNA methylation data derived from whole blood. Incorporating DNA methylation and gene expression data from different tissue types should therefore improve this analysis pipeline’s potential to help elucidate epigenetic mechanisms in disease, as both are known to be tissue-specific. This is the phenomenon whereby a gene’s function may be restricted to certain tissue types that may be relevant for a given disease.^54^ This is important as DNA methylation data derived from cord blood may only be acting as a proxy for a more relevant tissue type. For example, in this study this could be lung tissue given the associations we detected with measures of lung function. In-depth tissue-specific evaluations of the association signals detected in whole blood would therefore be worthwhile once such datasets concerning DNA methylation become available.^55^ Added complexities (such as multiple testing) when analysing various tissue types will need to be carefully considered in these study designs. However, as sample sizes for publicly available molecular datasets increase, so too will the necessary power required to detect associations similar to the two CpG sites highlighted in our study. This should facilitate mechanistic insight into the causal pathway between risk factors and later life outcomes, as well as disentangling co-expression between neighbouring genes, which is another current limitation in the field when evaluating signals. However, in the meantime there is increasing evidence that mQTL derived from blood act as a surprisingly adequate proxy for other tissue types.^56^

The approach demonstrated by this study should prove valuable in prioritizing genes to investigate the biological mechanisms that may explain associations between modifiable risk factors and disease susceptibility. The rewards for this challenging endeavour are promising, as it will allow us to use epigenetic factors as an indicator for early disease prognosis and subsequently improve health care and treatment.

## Funding

This work was supported by the Integrative Epidemiology Unit which receives funding from the UK Medical Research Council and the University of Bristol (MC_UU_00011/1 and MC_UU_00011/5). This work was also supported by CRUK (grant number C18281/A19169) and the ESRC (grant number ES/N000498/1). T.G.R. is a UKRI Innovation Research Fellow (MR/S003886/1). G.H. is supported by the Wellcome Trust [208806/Z/17/Z]. 

## Supplementary Material

dyz119_Supplementary_Material
