# Supplementary material for: An integrative approach to detect epigenetic mechanisms that putatively mediate the influence of lifestyle exposures on disease susceptibility
Source: Int J Epidemiol. 2019 Jun 29;48(3):887–98. doi: 10.1093/ije/dyz119 (PMC6659375; doi:10.1093/ije/dyz119)
Supplement: dyz119_Supplementary_Material [file dyz119_supplementary_material.zip › dyz119-suppl_data/Supplementary Material.docx]

## Methylation assays

DNA samples were bisulfite treated using the Zymo EZ DNA Methylation^TM^ kit (Zymo, Irvine, CA). The Illumina HumanMethylation450 BeadChip (HM450k) was used to measure methylation across the genome and the following arrays were scanned using Illumina iScan, along with an initial quality review using GenomeStudio. A purpose-built laboratory information management system (LIMS) was responsible for generating batch variables during data generation. LIMS also reported quality control (QC) metrics for the standard probes on the HM450k for all samples and excluded those which failed QC. Data points with a read count of 0 or with low signal:noise ratio (based on a p-value > 0.01) were also excluded based on the QC report from Illumina to maintain the integrity of probe measurements. Methylation measurements were then compared across timepoints for the same individual and with SNP-chip data (HM450k probes clustered using k-means) to identify and remove sample mismatches. All remaining data from probes was normalised with the Touleimat and Tost(1) algorithms using R with the wateRmelon package(2). This was followed by rank-normalising the data to remove outliers. Potential batch effects were removed by regressing data points on all covariates. These included the bisulfite-converted DNA (BCD) plate batch and white blood cell count which was adjusted for using the *estimateCellCounts* function in the minfi Bioconductor package(3).

## Genotyping assays

Genotype data were available for all ALSPAC individuals enrolled in the ARIES project, which had previously undergone quality control, cleaning and imputation at the cohort level. ALSPAC offspring selected for this project had previously been genotyped using the Illumina HumanHap550 quad genome-wide SNP genotyping platform (Illumina Inc, San Diego, USA) by the Wellcome Trust Sanger Institute (WTSI, Cambridge, UK) and the Laboratory Corporation of America (LCA, Burlington, NC, USA). Samples were excluded based on incorrect sex assignment; abnormal heterozygosity (<0.320 or >0.345 for WTSI data; <0.310 or >0.330 for LCA data); high missingness (>3%); cryptic relatedness (>10% identity by descent) and non-European ancestry (detected by multidimensional scaling analysis). After QC, 500,527 SNP loci were available for the directly genotype dataset. Following QC the final directly genotyped dataset contained 526,688 SNP loci.

## Imputation

Genotypes with MAF > 0.01 and Hardy-Weinberg equilibrium P > 5×10^-7^ were phased together using ShapeIt (version 2, revision 727)(4) and imputed using the 1000 Genomes reference panel (phase 1, version 3, phased using ShapeIt version 2, December 2013, using all populations) using Impute (v2.2.2)(5). After imputation dosages were converted to bestguess genotypes and filtered to only keep variants with an imputation quality score ≥ 0.8. The final imputed dataset used for the analyses presented here contained 8,074,398 loci.

1. Touleimat N, Tost J. Complete pipeline for Infinium(®) Human Methylation 450K BeadChip data processing using subset quantile normalization for accurate DNA methylation estimation. Epigenomics. 2012;4(3):325-41.

2. Pidsley R, Y Wong CC, Volta M, Lunnon K, Mill J, Schalkwyk LC. A data-driven approach to preprocessing Illumina 450K methylation array data. BMC Genomics. 2013;14:293.

3. Jaffe AE, Irizarry RA. Accounting for cellular heterogeneity is critical in epigenome-wide association studies. Genome biology. 2014;15(2):R31.

4. Delaneau O, Howie B, Cox AJ, Zagury JF, Marchini J. Haplotype estimation using sequencing reads. American journal of human genetics. 2013;93(4):687-96.

5. Howie BN, Donnelly P, Marchini J. A flexible and accurate genotype imputation method for the next generation of genome-wide association studies. PLoS genetics. 2009;5(6):e1000529.
