# Supplementary figures and images for: An integrative approach to detect epigenetic mechanisms that putatively mediate the influence of lifestyle exposures on disease susceptibility
Source: Int J Epidemiol. 2019 Jun 29;48(3):887–98. doi: 10.1093/ije/dyz119 (PMC6659375; doi:10.1093/ije/dyz119)

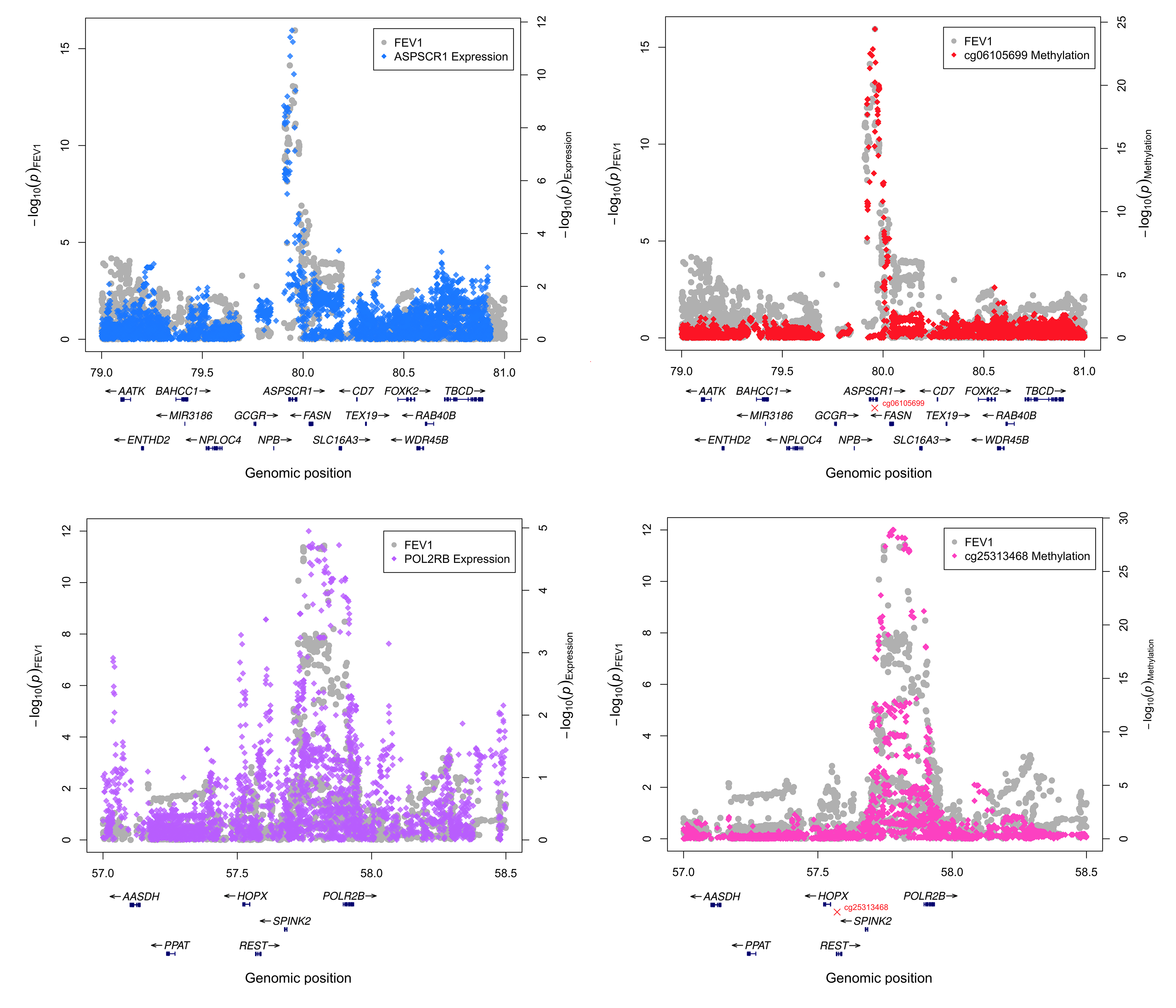

Supplement: dyz119_Supplementary_Material [file dyz119_supplementary_material.zip › dyz119-suppl_data/ije-2018-09-1155-File008.png]

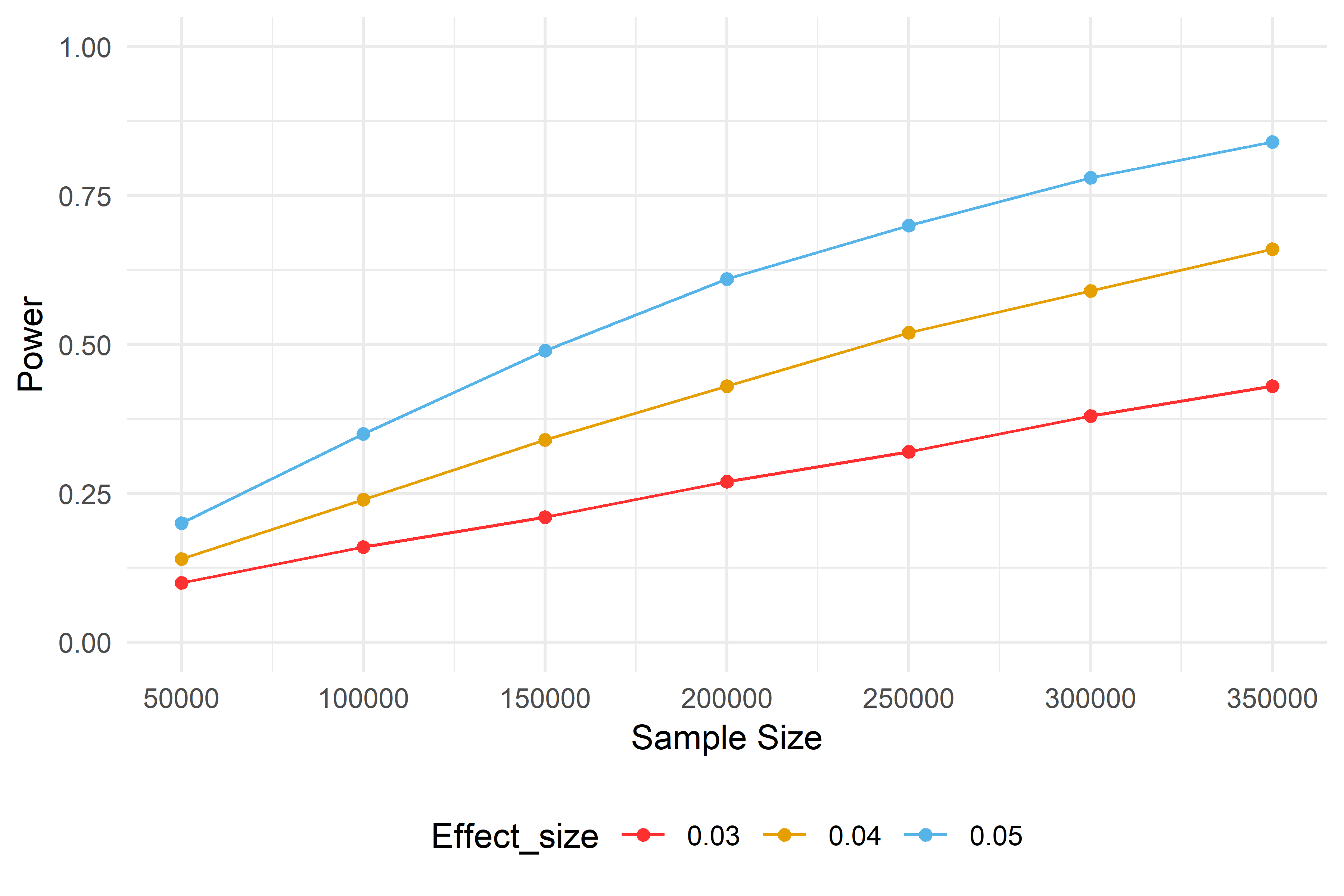

Supplement: dyz119_Supplementary_Material [file dyz119_supplementary_material.zip › dyz119-suppl_data/ije-2018-09-1155-File009.png]
